# The critical need to develop tools assessing cerebellar reserve for the delivery and assessment of non-invasive cerebellar stimulation

**DOI:** 10.1186/s40673-020-00126-w

**Published:** 2021-01-04

**Authors:** Mario Manto, Shinji Kakei, Hiroshi Mitoma

**Affiliations:** 1 Unité des Ataxies Cérébelleuses, Service de Neurologie, CHU-Charleroi, Charleroi, Belgium; 2 Service des Neurosciences, Université de Mons, Mons, Belgium; 3 Tokyo Metropolitan Institute of Medical Science, Tokyo, Japan; 4 Department of Medical Education, Tokyo Medical University, Tokyo, 160-0023 Japan

**Keywords:** Cerebellum, Reserve, Atrophy, tDCS, TMS, Non invasive stimulation

## Abstract

Non-invasive cerebellar stimulation (NICS) aims to modulate cerebello-cerebral loops and cerebro-spinal loops, both for research and clinical applications. It is of paramount importance to establish and validate morphological and functional tools to quantify cerebellar reserve, defined as the capacity for restoration and compensation to pathology of the cerebellum. Using NICS without efforts to estimate cerebellar reserve will end up in conflicting results due to the very high heterogeneity of cerebellar disorders encountered in daily practice.

Non-invasive cerebellar stimulation (NICS) is growingly applied worldwide, both in research laboratories and in clinical settings, with the goal of modulating cerebello-cerebral loops and cerebro-spinal loops [1]. There are several reasons for the increasing interest of the scientific community in NICS, making of the cerebellum a potentially ideal target in terms of investigations/therapeutic purposes in central nervous system (CNS) disorders [2–6]:

- the anatomical location of the cerebellum immediately below the skull
- the anatomical organization of the cerebellar circuitry into a cerebellar cortex at the periphery, surrounding white matter in which cerebellar nuclei are embedded
- the very high concentration of neurons in the cerebellar cortex
- the organization of the cerebellum into modules which are defined as conglomerates of several non-adjacent parasagittal bands of Purkinje neurons projecting to specific areas and gating segregated projections from the inferior olive
- the redundant and stereotypical architecture of the cerebellar cortex
- the multiple forms of plasticity occurring in the cerebellar circuitry at the level of parallel fibers/Purkinje neurons and at the level of interneurons of the cerebellar cortex
- the implication of the cerebellum in numerous cerebello-cerebral networks and cerebello-spinal networks
- the multiple roles of the cerebellum in motor control, cognitive operations and social/affective regulation, suggesting the NICS might be used not only in motor disorders (essential tremor, dystonia, Parkinson’s disease, cerebellar disorders) and in neurorehabilitation (stroke), but also in neuropsychiatric disorders for which a dysfunction or lesions of the cerebellum are suspected (schizophrenia, bipolar disorders, major depressive disorders, generalized anxiety disorder). Neurodevelopmental disorders affecting social skills such as autism/autism spectrum disorders are potential candidates also.
- the high responsiveness of the cerebellar cortex to electrical/magnetic stimuli.

NICS techniques gather tDCS (transcranial direct current stimulation), tACS (transcranial alternating current stimulation) and TMS (transcranial magnetic stimulation). tDCS and tACS modulate neuronal excitability but cannot induce directly action potentials. TMS (single pulse or repetitive TMS rTMS) may not only tune neuronal excitability, but can also trigger action potentials according to the intensity of the pulses applied. Both techniques (application of current and magnetic fields) have demonstrated a high safety profile for neurological disorders.

NICS primarily targets cerebellar cortex, which is composed of the molecular layer externally, the Purkinje cell layer and the granule cell layer internally. Currently, it is assumed that NICS modifies the cerebellum-brain inhibition (CBI), a mechanism by which the Purkinje neurons of the cerebellar cortex inhibit cerebellar nuclei, which themselves excite remote sites such as thalamic nuclei or brainstem nuclei [6]. Increasing the excitability of the Purkinje neurons results in increased inhibition of cerebellar nuclei (enhanced CBI). Furthermore, the recent discovery of disynaptic loops between striatum and cerebellum has opened an entire novel field of research in disorders such as dystonia or Parkinson’s disease, including for neuromodulation of the cerebellum [2, 7, 8].

Cerebellar ataxias (CAs) represent a group of heterogeneous disorders (vascular disease, trauma, local tumors, immune ataxias, genetic ataxias, …), both in terms of phenotypes, mechanisms of diseases and progression of symptoms [9]. Clinically, patients show various combinations of a cerebellar motor syndrome (CMS: involvement of lobules I-VI and VIII), a cerebellar vestibular syndrome (CVS: lobules V-VII, IX-X) and a Schmahmann/cerebellar cognitive affective syndrome (CCAS: lobules VI-IX) [10]. Despite modelling studies which provide estimations of the areas of the cerebellum which can be targeted [11], there are several unsolved issues regarding NICS and cerebellar ataxias:

- the montage and intensity/density of current applied: it remains unclear which is the best montage that should be used according to the disease
- the duration of sessions and their frequency of administration
- the anatomical variability (skull, cerebellar anatomy) between patients
- the role of NICS on the electrical properties of the CSF itself
- the quantification of the spreading effect (through the skin and skull, on remote brain networks)
- how NICS influences physiotherapy or rehabilitation procedures which are often proposed to patients
- which drugs complement or antagonize the effects of NICS and vice-versa.

Several studies are ongoing to assess the efficacy of NICS in CAs. We would like to underline a critical point, which is the notion of cerebellar reserve. Cerebellar reserve is defined as the capacity for restoration and compensation to pathology of the cerebellum [12]. Brain reserve (this applies also to cerebellar reserve) correlates with the number of remaining intact/undamaged neurons and synapses, thus being morphological and quantitative in nature. It is critical to introduce NICS during a period where cerebellar reserve is preserved. A typical example is cerebellar atrophy observed in degenerative CAs. Figure 1 illustrates various stages of cerebellar atrophy [13]. Once a severe atrophy is installed, the likelihood that NICS will be active on the cerebellar circuitry is extremely low, since most of the neurons/glial cells have disappeared.

**Fig. 1:**
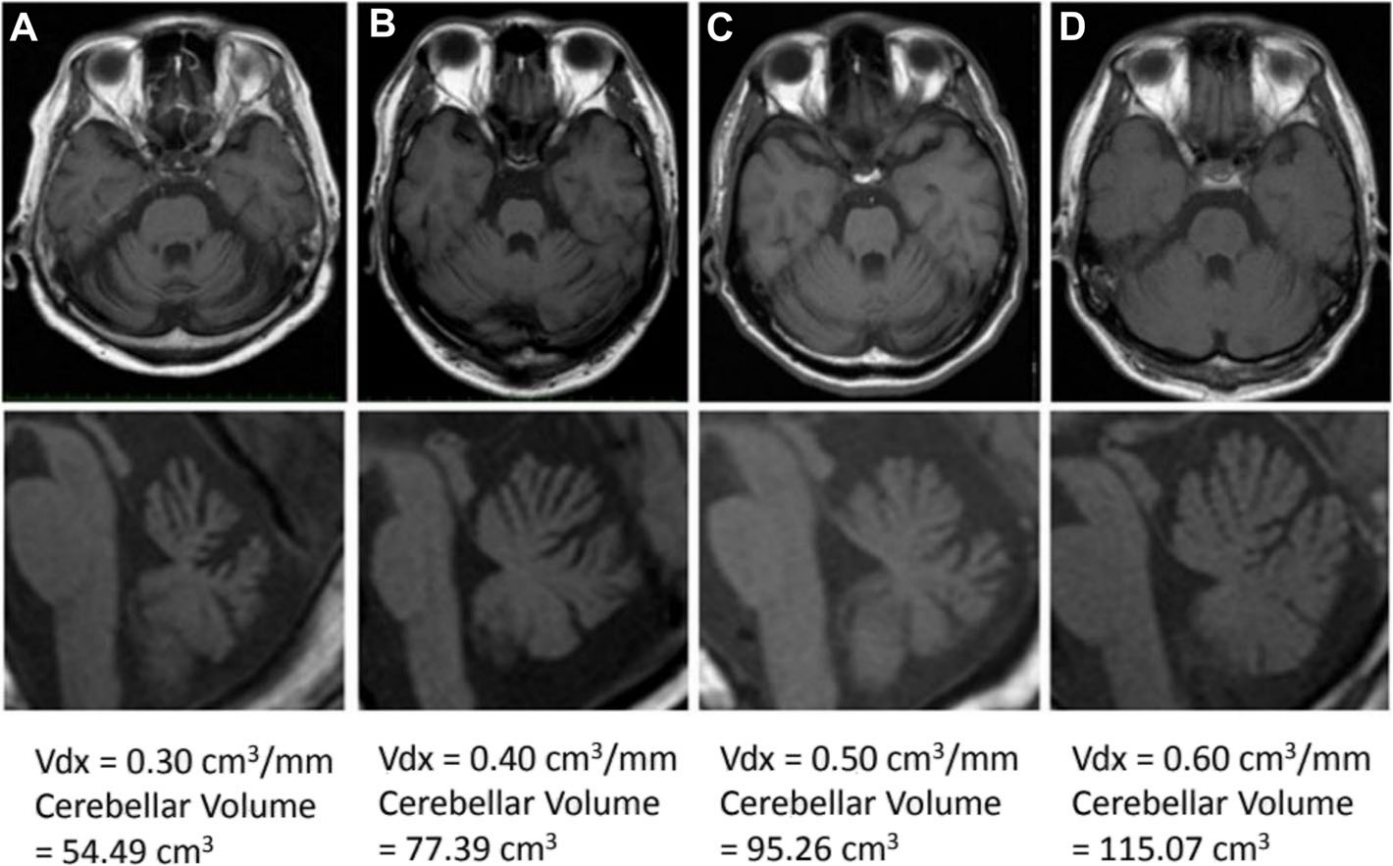
Various degrees of cerebellar atrophy are illustrated. From the most severe atrophy (**a**) to the most moderate (**d**). From: Cerebellum Ataxias 2017 Jun 29;4:9

Cerebellar reserve is both structural and functional [12]. Neuroimaging tools such as structural MRI, fMRI and diffusion tensor imaging (DTI) studies allow the estimation of the morphological aspects of the cerebellar reserve. For instance, volumetry of white and gray matter support the contribution of the cerebellum in cognitive tasks [14]. There is an urgent need to define which tasks should be selected for fMRI assessments of the cerebellar reserve (a consensus is required on the motor tasks, cognitive tasks, social/affective tasks which need to be applied). Furthermore, novel tools should be developed to quantify the cerebellar reserve from a functional standpoint. First, since effects of stimulation on the excitability of the cerebral cortex or the spinal cord reflect the degree of the neural preservation at the stimulation site, robust parameters complementing CBI are missing. Second, EMGs with natures of predictive activation could be indices for cerebellar control using the internal forward model, which is now considered as the most relevant theory for CAs [15]. Tailoring treatment to a particular patient requires defining in particular the extent of preservation of the cerebellar motor reserve. Biological tests (blood, CSF, other fluids such as tears or saliva) used as biomarkers may be useful also, for instance biochemical tests in CAs due to a metabolic defect or dosage of markers of neuroaxonal lesions such as neurofilaments [16]. The community might end up with negative or conflicting results for studies using NICS in CAs if the concept of cerebellar reserve is skipped and patients in whom the cerebellar reserve is severely affected are gathered with patients in whom the cerebellar reserve is relatively spared. Guidelines regarding simultaneous inclusion of pauci-symptomatic patients with patients with severe symptoms are missing, as well as safety datas in ataxic children and identification of the most relevant endpoint in studies addressing short-term/middle term/long term effects considering that CAs may be progressive degenerative disorders. A stratification of patients based on the quantification of the cerebellar reserve is warranted.

In conclusion, the cerebellum has a unique anatomical configuration and location. NICS has a strong feasibility and good safety profile. There is an urgent need to standardize protocols and plan large scale sham-controlled double-blind trials to convince health authorities, taking into account the degree of the cerebellar reserve especially in patients with cerebellar atrophy. Research is required to develop validated morphological and functional tools quantifying the cerebellar reserve with accuracy. Computational models are useful to optimize the identification of lobules whose activity is changed by NICS and understand how the electric field strength impacts on electric field distribution [11]. There is a requisite to elucidate which forms of cerebellar plasticity are modulated and whether NICS changes plasticity in remote regions of the brain involved in motor, cognitive, social/affective tasks such as cerebral cortex, limbic system and basal ganglia. There is also a necessity to elucidate which cerebellar modules are tuned by NICS [17], to which extent and for how long. Furthermore, there is a requirement to clarify the best method to modulate cerebellar networks for (a) disorders affecting primarily the cerebellar circuitry, and (b) disorders affecting remote regions and for which a neuromodulation of the cerebellum is expected to improve symptoms. Fitting the protocols to address inter-individual differences should be considered given the very high heterogeneity of cerebellar disorders and the need to complement pharmacotherapies [18]. We miss large studies combining clinical, electrophysiological, behavioral and neuroimaging techniques and we should consider that different symptoms might require different treatment approaches, because of different mechanisms behind cardinal symptoms [19]. In particular, therapy of cerebellar dysmetria might be distinct from therapy of cerebellar tremor. A symptom-based approach of NICS should thus be evaluated.

## Data Availability

Not applicable.

## Abbreviations

NICS: Non-invasive cerebellar stimulation
CNS: Central nervous system
tDCS: Transcranial direct current stimulation
tACS: Transcranial alternating current stimulation
TMS: Transcranial magnetic stimulation
CBI: Cerebellum-brain inhibition
CMS: Cerebellar motor syndrome
CVS: Cerebellar vestibular syndrome
CCAS: Schmahmann/cerebellar cognitive affective syndrome
CAs: Cerebellar ataxias
fMRI: Functional magnetic resonance imaging
DTI: Diffusion tensor imaging
